# COVID-19 and Upper Gastrointestinal Bleeding; Etiology, Risk Factors, and Outcomes: A Case-Control Study

**DOI:** 10.34172/mejdd.2025.406

**Published:** 2025-01-31

**Authors:** Sara Shafieipour, Mohammad Rezaei Zadeh Rukerd, Niloofar Farsiu, Mohsen Nakhaie, Samaneh Jahangiri, Maysam Yousefi, Hanieh Mirkamali, Aryan Mohamadinezhad

**Affiliations:** ^1^Gastroenterology and Hepatology Research Center, Institute of Basic and Clinical Physiology Sciences, Kerman University of Medical Sciences, Kerman, Iran; ^2^Clinical Research Development Unit, Afzalipour Hospital, Kerman University of Medical Sciences, Kerman, Iran; ^3^Research Center of Tropical and Infectious Diseases, Kerman University of Medical Sciences, Kerman, Iran; ^4^Physiology Research Center, Institute of Neuropharmacology, Kerman University of Medical Sciences, Kerman, Iran

**Keywords:** COVID-19, Gastrointestinal bleeding, Risk factor, Outcome, Iran

## Abstract

**Background:**

COVID-19-associated gastrointestinal (GI) symptoms are often self-limiting; however, gastrointestinal bleeding (GIB) is a critical complication in patients with COVID-19. The present study investigates the etiology, risk factors, esophagogastroduodenoscopy (EGD) findings, and outcomes associated with upper gastrointestinal bleeding (UGIB) in hospital inpatients with COVID-19.

**Methods:**

In this retrospective case-control study, 127 patients with COVID-19 in Kerman, Iran, were diagnosed using reverse transcription polymerase chain reaction (RT-PCR) and subsequently divided into case and control groups from January 2022 to July 2022.

**Results:**

This study evaluated 64 patients with COVID-19 with UGIB and 63 patients without. The case group reported previous history of GIB and cirrhosis at 17.2% and 12.5%, respectively (*P*=0.001 and *P*=0.01). Melena (37.5%) and peptic ulcer (21.87%) were the most common UGIB symptom and EGD findings, respectively. In the comparison of the case group with the control group, the duration of the patient’s stay in the intensive care unit (ICU) (11.58±1.13 vs. 8.29±1.06 days), the need for invasive mechanical ventilation (IMV) (17.2% vs. 8.1%), and the mortality rate (26.6% vs. 18.9%) were recorded (*P*=0.03, 0.124, and 0.07, respectively).

**Conclusion:**

Patients with COVID-19 and UGIB have a more prevalent ICU stay compared with those without. Melena and peptic ulcer were the most common presentations and EGD findings in these patients. Additionally, liver cirrhosis and a history of previous GIB increased the risk of GIB in patients with COVID-19.

## Introduction

 Human coronaviruses were first identified in the 1960s; some cause mild infection, while others, such as the severe acute respiratory syndrome coronavirus (SARS-CoV)-1 and SARS-CoV-2, can be severe and fatal.^1,2^ SARS-CoV-2 is the cause of COVID-19, which started in December 2020 in Wuhan, China, and was declared a new pandemic by the World Health Organization (WHO) on March 11, 2020.^3,4,5^ COVID-19 has infected more than 79 million patients and caused 1.7 million fatalities globally. COVID-19 impacts the respiratory system as well as other extrapulmonary organs, such as the central nervous and gastrointestinal (GI) systems.^2,3,4,6^

 SARS-CoV-2 is an enveloped, non-segmented positive-sense, and single-stranded ribonucleic acid (RNA) that enters the host cells through angiotensin-converting enzyme 2 (ACE2) receptors, expressed in various organs, including the intestines.^7,8^ ACE2 is extensively expressed in the GI tract, particularly on the brush borders of enterocytes in the gut epithelium, with levels approximately 100 times more than in alveolar epithelial cells.^2,9,10^ SARS-CoV-2 can be identified in the esophagus, gastric lamina propria or enterocytes, duodenum, and rectum.^2,11,12^ These findings indicate that GI tract infection may play a major role in COVID-19 transmission.^13^ Additionally, recent literature highlights the relevance of the gut–lung axis in COVID-19, suggesting that GI symptoms may arise as a response to respiratory infections, thereby emphasizing the need to further explore the underlying mechanisms contributing to GI complications.^14^

 GI tract involvement during COVID-19 can be secondary to direct viral injury or immune response, which can cause modification of intestinal permeability, imbalance of intestinal secretions, and activation of the enteric nervous system.^15^ The onset of GI manifestations is variable; although GI symptoms can present at the start of the disease, they typically develop later or during hospitalization.^2,15^ Variations in GI manifestations and onset time may be due to multiple factors, such as genetics, geographical differences, and patient’s medical histories.^2,16^

 COVID-19-associated GI manifestations, reported in 11.4%-66-1% of the patients, are primarily self-limiting and often include loss of appetite, nausea, vomiting, diarrhea, and abdominal pain.^2,4,17-19^ One of the most severe GI manifestations in patients with COVID-19 is GI bleeding, which is more lethal than other GI symptoms, with an incidence rate of 2%-3% in hospital inpatients and even more in ICU patients.^15,20,21^ Patients with COVID-19 have an increased risk of acute gastrointestinal bleeding (GIB) compared with healthy individuals.^20,22,23^ The increased risk of acute upper GI bleeding (UGIB) and lower GI bleeding (LGIB) in patients with COVID-19 can be due to conditions such as esophagitis, gastritis, peptic ulcers, and ischemic or hemorrhagic colitis, which can be exacerbated by COVID-19 treatments, including corticosteroids, anticoagulation, tocilizumab, and the need for mechanical ventilation.^2,24-27^ Nevertheless, conservative management has been proven to be successful in the management of COVID-19-associated GIB, with guidelines recommending esophagogastroduodenoscopy (EGD) evaluation within 24 hours of symptoms onset.^28-31^

 Multiple studies have indicated that patients with COVID-19 with GI symptoms have a higher viral load and shedding, greater incidence of disease progression to severe forms, increased mortality rate, and longer hospital stays compared with those without GI symptoms.^2,17,32-39^ Contrastingly, some studies discovered that patients with GI symptoms had a similar or lower risk of mechanical ventilation and mortality than those with COVID-19.^13,40^ This study aimed to clarify the etiology, risk factors, and outcomes of UGIB during COVID-19 among patients in Kerman, Iran.

## Materials and Methods

###  Study Design and Setting

 This retrospective case-control study was conducted on 127 hospital inpatients with COVID-19, both with or without UGIB, to determine the etiology of UGIB and its relationship with disease prognosis. The present study was conducted at Afzalipour Hospital, Kerman University of Medical Sciences, a large tertiary center in Iran, from January 2022 to July 2022, based on the STROBE guideline.^41^ The inclusion criteria for the case group were as follows: patients older than 18 years old with positive reverse transcription polymerase chain reaction (RT-PCR) test for COVID-19 and presented with UGIB. The inclusion criteria for the control group were identical to those of the case group, except that they did not present with UGIB. Exclusion criteria comprised patients with incomplete information on the questionnaire or positive RT-PCR test for COVID-19 after EGD.

## Outcomes and variables

 In this study, enrolled patients with positive PCR tests for COVID-19 were divided into two groups: 64 patients with UGIB in the case group, while the control group included 63 patients without UGIB.

 The questionnaire collected patient information, including demographic variables, background diseases, the need for invasive mechanical ventilation (IMV), intensive care unit (ICU) admission, and discharge status.

###  Statistical Analysis

 Patients’ data were analyzed using IBM SPSS software version 26 (SPSS Inc., Chicago, IL). The Kolmogorov-Smirnov test was used to check the normality of the data. Then, parametric and non-parametric tests were applied to analyze the normally and abnormally distributed data, respectively. Categorical variables are reported as numbers and percentages, while continuous variables are presented as mean ± standard deviation (SD). The Chi-square test and Pearson’s correlation coefficient were used for analytical statistics of qualitative and quantitative variables, respectively. Non-parametric tests, including Fisher’s test and Spearman’s correlation coefficient, are also applied for variables without normal distribution. The significance level is set at *P* value < 0.05.

## Results

###  Demographic Characteristics 

 A total of 127 patients were enrolled in this study. The case group had 64 patients with a mean age of 59.64 ± 2.57 years, and the control group had 63 patients with a mean age of 53.62 ± 1.99 years. In the case group, 33 (51.5%) were male and 31 (48.5%) were female, while in the control group, 32 (50.7%) were male patients, and 31 (49.3%) were female (*P* = 0.82). Laboratory findings of both groups at the time of admission and during hospitalization are represented in Table 1. Laboratory results showed significant differences between the two groups in hemoglobin (Hb) levels (*P* = 0.001) and activated partial thromboplastin time (PTT) (*P* = 0.006).

**Table 1:** Laboratory results of the case and control groups at the time of admission

	**Case group**		**Control group**		**Normal range**	**Units**	***P*** ** Value**
	**Mean**	**SD**	**Mean**	**SD**			
WBC	9.98	1.29	7.45	0.52	4.5-11.0 × 10^9^	Cell/L	0.08
Hb	10.75	0.4	12.12	0.28	M:13.2-16.6 W:11.6-15	g/dL	0.001
Plt	207.63	14.58	190.81	12.16	150000-450000	Cell/*µL*	0.69
AST	53.07	8.27	48.72	3.99	8-33	U/L	0.64
ALT	39.41	4.34	40.05	6.42	4-36	U/L	0.17
ALK	271.33	29.29	237.95	13.92	44-147	U/L	0.30
INR	1.24	0.04	1.32	0.12	< 1.1	-	0.54
PTT	35.5	1.24	46.3	3.67	25-35	Second	0.006
Urea	73.52	8.45	55.36	11.8	6-24	mg/dL	0.30
Cr	1.87	0.35	1.41	0.17	0.7-1.3	mg/dL	0.26
Albumin	3.17	0.12	3.32	0.12	3.4-5.4	g/dL	0.49

WBC: White blood cells; Hb: Hemoglobin; Plt: Platelets; AST: Aspartate aminotransferase; ALT: Alanine aminotransferase; ALK: Alkaline phosphatase; INR: International Normalized Ratio; PTT: Partial thromboplastin time; Cr: Creatinine.

 The underlying diseases of patients with COVID-19 were also recorded. Hypertension (31%), diabetes mellitus (26.2%), and ischemic heart disease (21.4%) were the most common underlying diseases, respectively. The previous history of GIB in the case and control groups was reported at 17.2% and 0%, respectively (*P* = 0.001). Cirrhosis was noted in 12.5% of the case group and 1.6% in the control group (*P* = 0.01).

###  Clinical Manifestations

 The common presentation of UGIB in the case group was melena (37.5%), fresh blood hematemesis (29.7%), coffee ground hematemesis (20.3%), and fresh rectal bleeding originating from above the ligament of Treitz (12.5%). Respiratory symptoms in patients with and without UGIB were reported in 12 and 48 patients, respectively (*P* = 0.001). In the case group, 42 (65.6%) patients reported an oxygen saturation level below 90% on room air, compared with 48 (76.19%) in the control group (*P* = 0.143)

###  EGD Findings

 The EGD investigation was performed to determine the GI changes in patients with UGIB (case group). The most frequent endoscopic finding was peptic ulcers (21.9%), while achalasia and gastric polyps were the least common (1.6% each). Other findings included normal results (20.3%), erosive gastritis (17.2%), gastric varices (7.8%), vascular lesions (7.8%), Mallory-Weiss syndrome (6.3%), esophageal varices (6.3%), esophageal ulcers (4.6%), and gastric cancer (3.1%).

###  Duration of ICU Admission and the Need for Invasive Mechanical Ventilation

 The need for IMV was observed in 17.2% of patients with COVID-19-associated GIB compared with 8.1% of those without GIB (*P* = 0.124). Additionally, the average duration of stay in the ICU was 11.58 ± 1.13 days for the case group and 8.29 ± 1.06 days for the control group (*P* = 0.03).

###  Patient’s Discharge Condition

 In evaluating the discharge conditions, 73.4% of patients with UGIB and 81.1% of patients without UGIB were discharged from the hospital, while the in-hospital mortality rate was 26.6% for patients with UGIB compared with 18.9% for those without bleeding (*P* = 0.07).

## Discussion

 Since the onset of the COVID-19 pandemic, UGIB has emerged as a significant complication in hospital inpatients, particularly those with severe disease. This case-control study compared 64 patients with COVID-19 and UGIB with 63 without, revealing that the case group was older and had a higher prevalence of previous GIB and cirrhosis. Melena was the most common clinical manifestation, and EGD investigations indicated a high rate of peptic ulcers in these patients. Furthermore, the case group had a significantly longer ICU stay, although the higher need for IMV and in-hospital mortality rates did not reach statistical significance.

 The retrospective cohort study by Alakuş and colleagues highlights that UGIB is relatively uncommon in patients with COVID-19, occurring in only 0.8% of those admitted to hospital. However, the findings underscore the significant mortality associated with UGIB, particularly in patients receiving steroid treatment, with non-survivors exhibiting a higher rate and duration of steroid use. The study suggests that most cases do not require endoscopic intervention and can be managed conservatively.^42^ Shafieipour and others conducted a retrospective study at Afzalipour Hospital in Kerman, Iran, over one year (April 2020 - March 2021) to assess the prevalence, risk factors, endoscopic findings, and outcomes of GIB in hospitalized patients with COVID-19. They found that 80 of 3,563 inpatients (2.24%) experienced GIB.^43^

 Mauro and colleagues reported that UGIB occurred in 0.47% of hospitalized patients with COVID-19, primarily those on anticoagulant therapy (78%). Peptic ulcer disease was the most common finding, with endoscopy performed within 24 hours in 48% of cases. Notably, mortality rates (21.7%) were linked to worsening COVID-19 infection, and outcomes did not significantly differ based on the timing of endoscopy.^44^

 Ashktorab and others conducted a systematic review of GIB in patients with COVID-19 from Western countries, analyzing 12 studies involving 808 patients. The overall GIB incidence was 0.06%. Compared with patients with COVID-19 and without GIB, those with GIB had higher mortality (25.4% vs. 16.4%). Melena was the most common presentation (47.5%), with peptic and esophageal ulcers as frequent EGD findings. Patients with GIB had higher rates of hypertension, liver disease, and cancer. Death was strongly linked to hypertension and hematochezia. GIB in patients with COVID-19 had similar incidence rates to the general population, but worse outcomes were observed.^45^ The systematic review and meta-analysis by Rathore and others underscore the prevalence and serious implications of UGIB occurs in 2.10% of patients with COVID-19 and is linked to higher severity (odds ratio = 3.52) and mortality (odds ratio = 2.16) compared with those without UGIB. Notably, the rebleeding rates (12.7%) further highlight the complications associated with UGIB in this population.^46^

 Merza and co-workers analyzed UGIB mortality trends in the United States using CDC WONDER data, revealing an increase from 3.3 per 100 000 in 2012 to 4.3 per 100 000 in 2021. They observed a significant year-on-year rise in mortality rates from 2012 to 2019, averaging 0.1 to 0.2 per 100 000, compared to a sharper increase of 0.4 to 0.9 per 100 000 from 2019 to 2021. These findings suggest a potential influence of the COVID-19 pandemic on UGIB mortality.^47^

 Cazacu and co-workers conducted a retrospective study on patients with UGIB admitted during the COVID-19 pandemic to assess outcomes compared with non-COVID-19 patients and a pre-pandemic cohort. Among 39 patients with UGIB and active COVID-19, the mortality rate was significantly higher at 58.97% (OR 9.04, *P* < 0.0001), primarily due to respiratory failure, with endoscopy performed in only half of the cases. UGIB admissions decreased by 23.7% during the pandemic, highlighting the increased mortality risk associated with COVID-19 in patients with UGIB, likely due to treatment delays.^48^

 The study by Rosevics and colleagues reveals a significant increase in urgent/emergency endoscopic procedures during the COVID-19 pandemic. Notably, the need for ICU admission and IMV was identified as a significant risk factor for UGIB in patients with COVID-19.^49^ Prasoppokakorn and others studied risk factors for UGIB in hospitalized patients with COVID-19 and the effectiveness of proton pump inhibitor (PPI) prophylaxis. Among 6,373 patients, 43 (0.7%) developed UGIB. Key findings included higher Glasgow-Blatchford scores and the absence of PPI use as significant risk factors. After PPI prophylaxis, UGIB incidence decreased slightly, with no active cases reported.^50^

 Our study had some limitations. Firstly, the low sample size may have resulted in some non-significant relationships going undetected, such as in-hospital mortality. Additionally, there was a lack of data on medication use, and the absence of EGD evaluation in the control group means that potential undiagnosed GI conditions cannot be ruled out. Further research is needed to clarify the etiology of UGIB in patients with COVID-19 and to explore preventive and management strategies to mitigate its impact on patients’ outcomes.
